# Anti-Myelin Oligodendrocyte Glycoprotein and Human Leukocyte Antigens as Markers in Pediatric and Adolescent Multiple Sclerosis: on Diagnosis, Clinical Phenotypes, and Therapeutic Responses

**DOI:** 10.1155/2018/8487471

**Published:** 2018-11-22

**Authors:** Maria P. Gontika, Maria C. Anagnostouli

**Affiliations:** ^1^Immunogenetics Laboratory of 1st Department of Neurology, Medical School of National and Kapodistrian University of Athens, Aeginition Hospital, Athens, Greece; ^2^Demyelinating Diseases Clinic, 1st Department of Neurology, Medical School of National and Kapodistrian University of Athens, Aeginition Hospital, Athens, Greece

## Abstract

Early-onset (pediatric and adolescent) multiple sclerosis (MS) is a well-established demyelinating disease that accounts for approximately 3-5% of all MS cases. Thus, identifying potential biomarkers that can reflect the pathogenic mechanisms, disease course and prognosis, and therapeutic response in such patients is of paramount importance. Myelin oligodendrocyte glycoprotein (MOG) has been regarded as a putative autoantigen and autoantibody target in patients with demyelinating diseases for almost three decades. However, recent studies have suggested that antibodies against MOG represent a distinct clinical entity of dominantly humoral profile, with a range of clinical phenotypes closely related to the age of onset, specific patterns of disease course, and responses to treatment. Furthermore, the major histocompatibility complex (MHC)—which has been regarded as the “gold standard” for attributing genetic burden in adult MS since the early 1970s—has also emerged as the primary genetic locus in early-onset MS, particularly with regard to the human leukocyte antigen (HLA) alleles DRB1⁎1501 and DRB1⁎0401. Recent studies have investigated the potential interactions among HLA, MOG, and environmental factors, demonstrating that early-onset MS is characterized by genetic, immunogenetic, immunological, and familial trait correlations. In this paper, we review recent evidence regarding HLA-genotyping and MOG antibodies—the two most important candidate biomarkers for early-onset MS—as well as their potential application in the diagnosis and treatment of MS.

## 1. Introduction

Multiple sclerosis (MS) is a chronic autoimmune demyelinating and neurodegenerative disease of the central nervous system (CNS), representing the most common cause of neurological disability among young adults. For decades, the pediatric and adolescent form of the disease constituted a controversial entity that often escaped diagnosis. Presently, early-onset (pediatric and adolescent) MS is a well-established demyelinating disease that accounts for approximately 3-5% of all MS cases [1–3].

Different mechanisms of demyelination, neurodegeneration, gliosis, and remyelination converge in various ways to create a unique clinical result for each patient with MS. Thus, identifying potential biomarkers that can reflect the pathogenic mechanisms, disease course and prognosis, and therapeutic response is of paramount importance. However, no studies to date have identified absolute surrogates for MS [4]. In this paper, we review recent evidence regarding human leukocyte antigen (HLA) genotyping and myelin oligodendrocyte glycoprotein (MOG) antibodies—the two most important candidate biomarkers for early-onset MS—as well as their potential application in the diagnosis and treatment of MS.

## 2. Main Text

### 2.1. MOG Antibody-Related Disorders: Phenotypical Spectrum, Prognosis, and Treatment

Although MOG comprises less than 0.05% of all CNS myelin proteins, it is localized on the outermost surface of the myelin sheath, making it an excellent antibody target [5]. The human antibodies (Abs) to MOG exhibit all the characteristics of pathogenic autoantibodies: they recognize MOG in its correct conformation, they are mostly of the complement fixing isotype IgG1, and they activate Ab-dependent cellular cytotoxicity, although their exact pathogenic role remains to be further clarified [5–7].

MOG antibodies are continually identified in a range of acquired demyelinating syndromes (ADS) in both adults and children. MOG antibodies are present in up to one-third of children with ADS, especially in patients who have experienced an acute demyelinating episode prior to the age of 10 years. Furthermore, previous studies have demonstrated a link between MOG antibodies and non-MS diagnoses [8–11].

While older reports provided controversial evidence, more recent studies have revealed that children with MOG antibodies can present with either a monophasic or multiphasic disease course (in up to 50% of cases), primarily depending on their age [9–14]. Previous research has indicated that patients with a monophasic disease course are more often younger and male. Moreover, in the majority of cases (50%), monophasic forms of the disease manifest as acute disseminated encephalomyelitis (ADEM), especially in patients under the age of 5 years [9, 12, 15, 16]. Other monophasic subtypes include monophasic neuromyelitis optica spectrum diseases (NMOSDs) accompanied by optic neuritis and/or transverse myelitis [9, 12, 17, 18], as well as clinically isolated syndromes [optic neuritis (ON), transverse myelitis (TM), cerebellitis, and brainstem disease], which present after puberty and do not confer risk factors for further MS-like episodes, such as positive oligoclonal bands (OCBs) or MS-like lesions on MRI [9, 10, 12, 16]. Interestingly, in patients with the monophasic subtype, anti-MOG titers tend to be transient and fall to undetected levels during the months following an acute episode [9, 12, 19]. In contrast, especially in older female patients with high and persistent MOG titers, different multiphasic subtypes have been identified, including multiphasic disseminated encephalomyelitis (MDEM); ADEM followed by relapsing episodes of ON (ADEM-ON); NMOSD; and relapsing, steroid-responsive ON [9, 12, 20–27]. Recently, MOG Abs have been detected in cases of NMDAR encephalitis, further expanding the phenotypic spectrum of the disorder [28, 29].

Only a very small proportion of both children and adults with MS present with MOG antibody seropositivity, possibly representing a distinct phenotype which could benefit from different treatment strategies. This is in accordance with biopsy findings in MOG antibody patients with acute demyelinating episodes, which reveal a lesion pattern similar to MS pattern II with demyelination and complement activation [9, 30–33]. Moreover, Hennes et al. suggested that a cutoff higher than previously anticipated (e.g., ≥1,280) can be used to increase the specificity for a non-MS disease course and facilitate the interpretation of MOG assay results [9].

The vast majority of recent studies suggest that persistently high-titer MOG Abs, but not their sole presence at onset, are associated with a high risk of relapse and that serial testing during and between clinical relapses could be reasonable for safe therapeutic decision-making, when attempting to predict the clinical course in MOG antibody positive patients [9, 11, 12, 34]. In general, patients with monophasic disease tend to exhibit more favorable outcomes (i.e., resolution of clinical and imaging abnormalities), which does not seem to be the case in patients with relapsing disease, who tend to exhibit a high relapse rate and progressive impairments [9, 12, 34, 35].

Paraclinical tests have also been associated with a distinct profile in patients with MOG antibody associated diseases. Cerebrospinal fluid (CSF) analysis in MOG antibody positive children is mandatory for detecting pleocytosis and the usual absence of OCBs (90% of cases), while double positivity for both MOG and anti-aquaporin 4 (AQP4) is rare in patients with NMOSD phenotypes and usually not statistically significantly different from healthy controls [8, 9, 12, 36]. MRI findings also vary among clinical phenotypes. In younger patients with an ADEM-like presentation, MRI findings are usually characterized by poorly demarcated and widespread lesions, sometimes with extensive myelin involvement including the conus. Such lesions exhibit no postcontrast enhancement and tend to resolve. In older patients, MRI findings can align with those of NMOSD, which is associated with extensive involvement of the optic nerves and periependymal areas as well as longitudinal extensive transverse myelitis (LETM). In such patients, lesions occasionally extend rostrally into the medulla and often the conus, although normal MRI findings are common in patients with pure relapsing ON [12, 16, 37]. The few MOG antibody seropositive children diagnosed with relapsing–remitting MS tend to exhibit a typical MS-like pattern with well demarcated periventricular and curved juxtacortical lesions involving U-fiber lesions, Dawson finger-type lesions, and short transverse myelitis [38].

In the acute phase, intravenous steroid treatment followed by* per os* tapering, intravenous immunoglobulin treatment, and plasmapheresis cycles appears to be associated with good responsiveness [12, 24, 37, 39]. However, the most effective treatments for relapsing disease remain to be clarified. Children with MOG antibody associated disorders generally did not benefit from the disease-modifying therapies commonly used in MS, in those rare cases that they have been employed, which also led to dramatic aggravation of symptoms in select cases [40–43]. B-cell-directed interventions (e.g., plasmapheresis and rituximab) have yielded positive results, along with certain immunosuppressive drugs (e.g., mycophenolate, azathioprine) [12, 44–46]. In their recent study of 102 children, Hacohen et al. reported that IV immunoglobulin maintenance therapy was the only intervention to significantly improve relapse rates and functional outcomes, expanding its well-known immunomodulatory effect in a probable dose-dependent manner [34].

### 2.2. HLA Alleles in Pediatric MS

Linkage studies in various populations have consistently demonstrated that the MHC and its polymorphisms represent the genetic locus most strongly linked to MS [47, 48]. In a recent collaborative European study, DRB1*∗*1501 (split of DR2) exhibited the strongest association with MS, along with DRB1*∗*0301 and DRB1*∗*1301, while HLA-A*∗*0201 has been shown to confer protection against MS [49].

Numerous studies have verified the association between DRB1*∗*15 and early-onset MS [50–52], supporting the notion that genetic contributions are fundamentally similar in both early-onset and adult MS. However, whether HLA-DRB1*∗*1501 by itself lowers the age at MS onset remains controversial, with several studies indicating that both genetic and environmental epistatic interactions may play a role [53–71]. Several hypotheses have been proposed in an effort to explain this discrepancy. Ramagopalan et al. observed that only maternally transmitted DRB1*∗*15 promotes lower age at MS onset, suggesting a possible parent of origin effect, while others have implicated relative fluctuations in vitamin D levels among different populations [72, 73].

In 2010, a remarkable DRB1-genotyping study in Australia became the first to demonstrate the significance of epistatic interactions at the HLA-DRB1 locus. Carriage of the DRB1*∗*1501 risk allele was not significantly associated with age at onset, while the DRB1*∗*0401 allele was associated with a reduced age at onset when combined with DRB1*∗*1501 [74].

In our recent study in the Greek population, we proposed that the DRB1*∗*03 allele may be associated with early-onset MS. However, further studies are required to verify this finding, as this allele has been associated not only with a presumably better MS prognosis, but also with NMO, a mainly humoral immunological entity [50].

In parallel with HLA studies, recent genome-wide association studies (GWAS) have provided evidence for more than 50 single-nucleotide polymorphisms (SNPs) of more modest effect that influence the risk of both adult MS and early-onset MS, further equalizing the genetic burden of these age groups [75] and suggesting possible clinical correlations [76]. In a 2017 study by Gianfrancesco et al., the authors analyzed 28 non-MHC mutations in 569 cases of early-onset MS. Despite the extensive literature regarding adult MS, they concluded that—while the generally observed higher burden did not reach statistical significance—the weighted genetic risk score of these mutations was significantly associated with pediatric-onset MS [77].

## 3. Discussion

Pediatric and adolescent MS is a well-defined clinical entity with established diagnostic criteria, whose early diagnosis and treatment may alter clinical outcomes in younger patients. In the context of the largely expanding therapeutic repertoire, the identification of genetic and antibody biomarkers may help guide diagnosis, predict disease course, and achieve targeted treatment. MOG antibodies and HLA alleles, either individually or via interactive mechanisms, have emerged as the two most promising candidates for such biomarkers.

MOG has been identified as a putative autoantigen and autoantibody target in patients with demyelination for almost three decades. However, only recently have cell-based assays (CBAs) and large-scale studies revealed its role in childhood and adolescent demyelination [23]. Earlier studies investigated MOG autoimmunity within the framework of specific demyelinating syndromes, including ADEM and pediatric MS, and while in ADEM cases the results were consistent, usually contradictory results were obtained in patients with CIS and an association with conversion to MS, especially before the introduction of CBAs [18, 78–90]. More recent evidence has demonstrated that antibodies against MOG may be associated with a clinical entity distinct from MS and AQP4-positive NMOSD [91–96]. This new entity, of dominantly humoral profile, is characterized by a range of clinical phenotypes closely related to the age of onset, with their own course, response to treatment, and prognosis, expanding the differential diagnostic work-up for patients with suspected demyelinating diseases. Thus, Hacohen et al. proposed a diagnostic algorithm for relapsing ADS in children, identifying five categories of relapsing DS (MS, anti-AQP4-positive NMOSD, MOG antibody associated disorders, and antibody-negative RDS) and highlighting the importance of anti-MOG testing in the differential diagnosis of MS [97]. Moreover, extended clinical and radiologic diagnostic criteria regarding the spectrum of MOG associated disorders have been recently proposed and although they could be adjusted on the basis of the evolving knowledge in this field, they could provide great assistance in everyday clinical practice [98–101].

In contrast, since its discovery and the early disease association studies of the 1970s, the MHC and its polymorphisms have represented the “gold standard” for attributing genetic burden in adult MS. Recently studies have further established the role of HLA-DRB1*∗*1501 in pediatric and adolescent, while the roles of HLA-DRB1*∗*0401 and HLA-DRB1*∗*03 remain to be clarified. Furthermore, HLA genotype may interact with MOG and environmental factors, especially viruses, which may explain in part the clinical diversity of MOG antibody associated disorders. In particular, HLA-DRB1*∗*0401, which appears to correlate with younger age at onset via epistatic interactions with HLA-DRB1*∗*1501, also appears to bind with high affinity to MOG epitopes in both familial patients with MS and asymptomatic relatives. These findings indicate that humoral immune reactivity against MOG is partially under the control of certain HLA class II alleles [102–107]. This observation may be helpful in guiding therapy, as the HLA-DRB1∗0401 allele is associated with a greater risk of developing neutralizing antibodies against interferon beta (IFN-*β*), which have been linked to poor therapeutic outcomes in adults [108]. Furthermore, in their recent breakthrough study, Morandi et al. investigated the potential interactions among MOG, HLA, and the Epstein–Barr virus (EBV), each of which is known to play a pathogenetic role in MS. Their findings demonstrated that EBV infection of B cells alters MOG processing, facilitating its cross-presentation to autoaggressive cytotoxic CD8 + T cells in an MHC-restricted manner, highlighting the interplay between genetic and biological factors in MS [109].

Abundant evidence supports the notion that MS is influenced by genetic, immunogenetic, immunological, and familial trait correlations.  Table 1 [12, 50–71, 74, 98–101, 110–117] summarizes the available data regarding the distribution of HLA alleles and MRI findings for five demyelinating syndromes: clinically definite MS, AQP4-positive NMOSD, MOG antibody associated disorders, and antibody-negative ADEM. As indicated in the table, there is an obvious lack of information regarding HLA genotyping in pediatric and adolescent ADS, despite the fact that primary results demonstrate clear genetic diversity. We strongly believe that larger HLA-genotyping studies regarding early-onset demyelinating disorders are necessary. Such studies should be conducted in various ethnic groups in order to clarify, replicate, and expand the available evidence.

## 4. Conclusions

HLA alleles and MOG antibodies have emerged as two major biomarkers of pediatric and adolescent MS and related demyelinating disorders. Each of these putative biomarkers exhibits a separate correlation with MS pathogenesis, clinical course, and treatment responses, suggesting that these factors interact to influence MS phenotypes and outcomes, an assumption further supported by recent evidence. Thus, further large-scale studies regarding HLA and MOG antibodies are required to verify and expand our knowledge of early-onset MS and to determine the appropriate biomarkers for distinct clinical phenotypes.

## Figures and Tables

**Table 1:** Summary of the available data regarding the distribution of HLA alleles and MRI findings for the four major pediatric demyelinating clinical entities, as they have emerged under the scope of the latest genetic, antibody, and imaging markers [12, 50–71, 74, 98–101, 110–117].

	**Clinically definite MS**	**AQP4 (+) NMOSD **	**MOG antibody associated disorders**	**ADEM Ab(-) **
**HLA allele **	HLA-DRB1*∗*1501 (Caucasian) HLA-DRB1*∗*0401 (Caucasian) HLA-DRB3*∗*02 & HLA-DRB1*∗*13 & HLA-DQB1*∗*03 (Korean)	HLA-DRB1*∗*03 (adult Caucasian) HLA-DRB1*∗*0501 (adult Japanese)	HLA-DRB1*∗*0401 (?)	HLA-DRB1*∗*01 & HLA-DRB1*∗*017 (Russian) HLA-DRB1*∗*1501 & HLA-DRB5*∗*0101 (Korean) HLA-DQB1*∗*0602& HLA-DRB1*∗*1501 & HLA-DRB1*∗*1503 (Brazilian) HLA-DRB1*∗*16 & HLA-DQB1*∗*05 (Caucasian adult)

**MRI **	Ovoid, well-defined lesions in at least two regions (periventricular, cortical, or juxtacortical U-fibers, infratentorial and spinal cord), Dawson fingers and black holes in T1, ring pattern of Gd enhancement	(1) Lesions (usually small & localized) involving the dorsal medulla and the periependymal surface of the ventricles; large, confluent, unilateral, or bilateral subcortical/deep white matter lesions and long lesions (>1/2 length) of the corpus callosum (2) LETM with probable rostral extension of the lesion into the brainstem (3) Unilateral or bilateral increased T2 signal or Gd enhancement within optic nerve or optic chiasm, >1/2 the distance from orbit to chiasm	(1) Longitudinally extensive spinal cord lesion (≥3 VS, contiguous) on MRI (so-called LETM) (2) Longitudinally extensive spinal cord atrophy (≥3 VS, contiguous) on MRI in patients with a history compatible with acute myelitis (3) Conus medullaris lesions, especially if present at onset (4) Longitudinally extensive optic nerve lesion (e.g., >1/2 of the length of the pre-chiasmal optic nerve, T2 or T1/Gd) (5) Perioptic Gd enhancement during acute ON (6) Normal supratentorial MRI in patients with acute ON, myelitis and/or brainstem encephalitis (7) Brain MRI abnormal but no lesion adjacent to a lateral ventricle that is ovoid/round or associated with an inferior temporal lobe lesion and no Dawson's finger-type or juxtacortical U fiber lesion (Matthews-Jurynczyk criteria) (8) Large, confluent T2 brain lesions suggestive of ADEM	Large, diffuse, poorly demarcated (>1 to 2 cm) lesions involving predominantly the cerebral white matter; deep gray matter lesions; T1-hypointense lesions in the white matter are rare

MS: multiple sclerosis, ADEM: acute disseminated encephalomyelitis, NMOSD: neuromyelitis optica spectrum disorders, MOG: myelin oligodendrocyte glycoprotein, LETM: longitudinally extensive transverse myelitis, Gd: gadolinium, VS: vertebral segments.

## Abbreviations

MS: Multiple sclerosis
MHC: Major histocompatibility complex
HLA: Human leukocyte antigens
MOG: Myelin oligodendrocyte glycoprotein
AQP4: Aquaporin-4
Abs: Antibodies
CSF: Cerebrospinal fluid
OCBs: Oligoclonal bands
ADS: Acquired demyelinating syndromes
ADEM: Acute disseminated encephalomyelitis
MDEM: Multiphasic disseminated encephalomyelitis
ADEM-ON: ADEM followed by optic neuritis
ON: Optic neuritis
TM: Transverse myelitis
NMO: Neuromyelitis optica
NMOSD: NMO spectrum diseases
GWAS: Genome-wide association studies
SNPs: Single-nucleotide polymorphisms
CBAs: Cell-based assays
IFN-*β*: Interferon beta
CNS: Central nervous system.
